# Microbiome Profiling of Enterotoxigenic Escherichia coli (ETEC) Carriers Highlights Signature Differences between Symptomatic and Asymptomatic Individuals

**DOI:** 10.1128/mbio.00157-22

**Published:** 2022-05-10

**Authors:** Ellen E. Higginson, M. Abu Sayeed, Joana Pereira Dias, Vignesh Shetty, Mamatha Ballal, Sunil Kumar Srivastava, Ian Willis, Firdausi Qadri, Gordon Dougan, Ankur Mutreja

**Affiliations:** a Cambridge Institute for Therapeutic Immunology and Infectious Disease (CITIID), Department of Medicine, University of Cambridge, Cambridge, United Kingdom; b International Centre for Diarrhoeal Disease Research Bangladesh, Dhaka, Bangladesh; c Enteric Diseases Division, Department of Microbiology, Kasturba Medical College, Manipal Academy of Higher Education, Manipal, India; d University of Delhi, Delhi, India; e EMBL-EBI, Hinxton, United Kingdom; f Translational Health Science and Technology Institute, Delhi-NCR, India; GSK Vaccines

**Keywords:** ETEC, microbiome, metagenomics

## Abstract

Enterotoxigenic Escherichia coli (ETEC) is an important cause of diarrhea in children in low- and middle-income countries (LMICs). However, large-scale pathogen burden studies in children have identified ETEC in the guts of both symptomatic patients and controls. The factors that influence this balance are poorly understood, but it is postulated that the gut microbiome may play a role in either resistance or progression to disease. In this study, we profiled the microbiomes of children and adults from Bangladesh who were asymptomatically or symptomatically infected with ETEC. Symptomatic patients had significantly higher numbers of sequenced reads mapping to both E. coli and two ETEC toxins, suggesting higher bacterial burden. They were also significantly more likely to be coinfected with enteroaggregative E. coli (EAEC) and had higher proportions of other *Gammaproteobacteria*, including Klebsiella, Salmonella, and Haemophilus. Colonization with ETEC was also associated with increased prevalence of antimicrobial resistance (AMR) genes, most notably those of the β-lactamase class. Taxonomic profiles were distinctly different between all groups in both species richness and composition, although the direction of these changes was different in adults and children. As seen previously, children with high E. coli burdens also had higher proportions of Streptococcus spp., while healthy children were more heavily colonized by *Bifidobacterium* spp. Our study provides insight into the microbiome changes that occur upon infection with ETEC in an endemic setting and provides rationale for future studies investigating how the microbiome may protect or predispose individuals to symptomatic infections with gastrointestinal pathogens.

## INTRODUCTION

Enterotoxigenic Escherichia coli (ETEC) is a common cause of watery diarrhea in low- and middle-income countries (LMICs). While normally described as the major cause of traveler’s diarrhea in visitors to these regions, ETEC also infects young children in areas of endemicity, causing an estimated 84.4 million diarrhea episodes per year worldwide (1). Although most people in these regions will generate immunity in childhood, this immune protection is not absolute, and adults may periodically endure symptomatic infections. From large-scale field studies, it is also clear that individuals can often carry ETEC without displaying significant clinical symptoms (2). Although it is not known what modulates the balance between symptomatic and asymptomatic infections, factors such as preexisting immunity and the composition of the microbiome may play a part.

In recent years, there has been increasing interest in understanding how the microbiome impacts human health. However, the majority of this microbial profiling has been performed on healthy adult individuals in developed countries and may not be generalizable or applicable to people in geographically distinct regions. Exemplar studies have found that the microbiomes of people in the United States and Europe are distinct from those in Africa and India (3–5). Some of this can be described by the *Prevotella*/*Bacteroides* gradient within the *Bacteroidetes* phylum, whereby *Bacteroides* predominates in Western populations, while *Prevotella* dominates elsewhere. *Bacteroidetes* are also more generally overrepresented in Western microbiomes, with *Firmicutes* being more common in other populations. This is also true in children, whereby children in Bangladesh were shown to have much lower levels of *Bacteroidetes* in total and higher microbial diversity than children in the United States (6). Similar patterns were also noted in adult U.S. travelers to Central America and India, with travelers having relatively more *Firmicutes* and fewer *Bacteroidetes* on return to the United States (7). These regional variations in microbiome composition are likely to be mediated by a multitude of factors, including diet, genetics, lifestyle, and the environment (4).

One environmental factor that is expected to have a major influence on gastrointestinal health is the interaction between the microbiome and gastrointestinal pathogens. Although several studies have looked at the impact of intestinal infection on the gut microbiome (8–13), due to differences in patient age, pathogen of interest, control groups and antibiotic therapy, as well as small patient sample numbers, there is no consensus on which aspects of the microbiome may be protective or endow colonization resistance and which might facilitate progression to disease. In a human ETEC challenge study, researchers identified several species that were correlated with progression to disease or resistance to infection, including several *Bacteroides* spp. which were overrepresented in volunteers who were protected from symptomatic infection (14). However, as this study was in healthy adults in the United States, it is not clear how relevant these findings are to populations where ETEC is endemic.

In our study we used metagenomic sequencing analysis to compare the gut microbiomes of adults and children in Bangladesh who were asymptomatically or symptomatically infected with ETEC. The whole-genome shotgun sequence data were interrogated to determine the burden of E. coli species, presence of ETEC toxins, antimicrobial resistance genes (ARGs), other bacterial pathogens, and overall microbiome composition.

## RESULTS

### Burden of E. coli in symptomatically and asymptomatically ETEC-infected individuals.

ETEC culture-positive stool samples were collected from 32 symptomatic and asymptomatic adults and children at the International Centre for Diarrhoeal Disease Research, Bangladesh (icddr,b) in Dhaka, Bangladesh. As controls, 16 ETEC culture-negative stool samples were collected from healthy adults and children in the same city. DNA was extracted from stool samples and shotgun sequenced using Illumina HiSeq technology. To determine the approximate ETEC load in each sample, sequenced reads were mapped to the O78 ETEC reference genome H10407. The percentage of reads that mapped to E. coli H10407 was then compared for each health status and age group.

**FIG 1:**
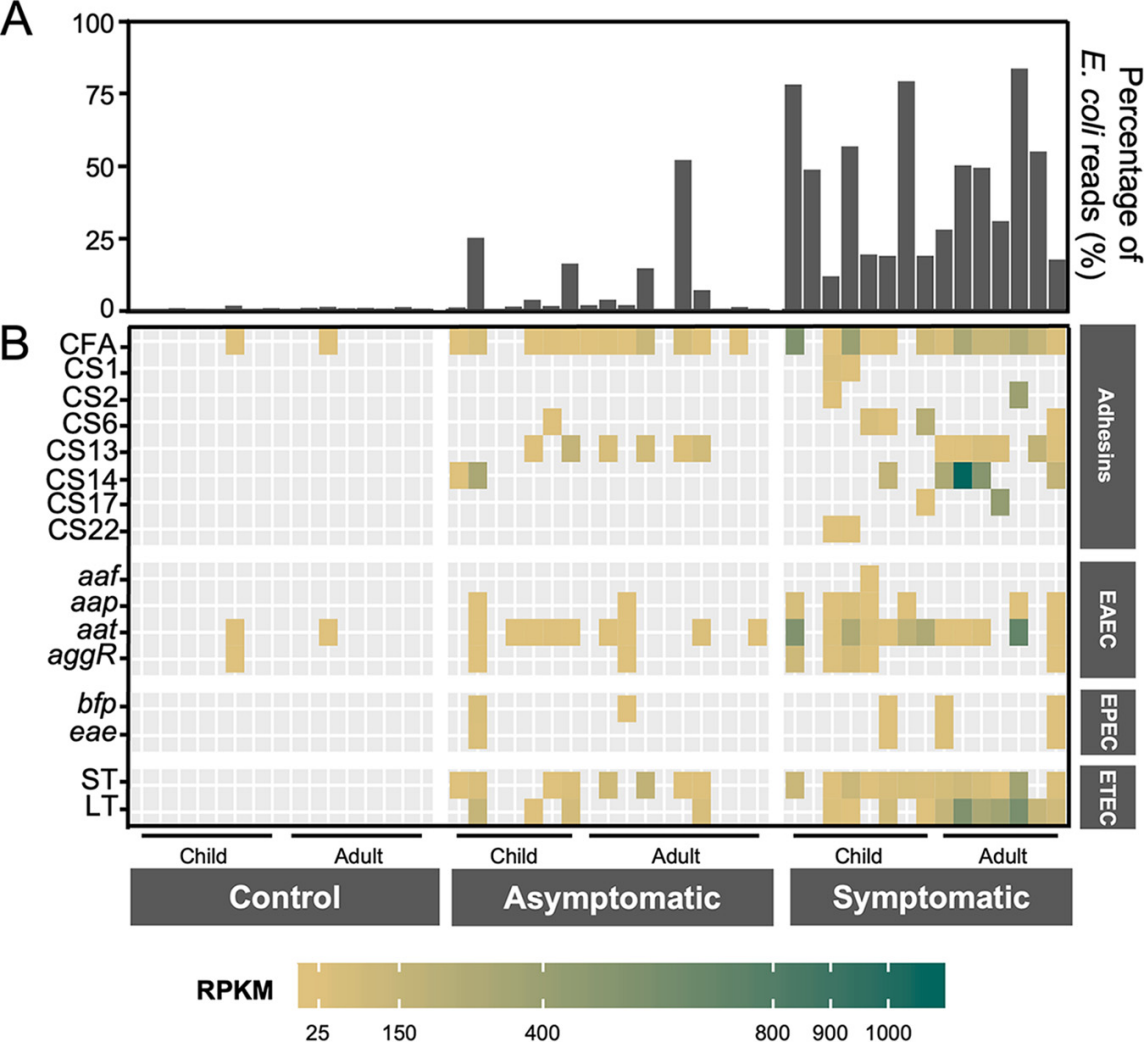
Detection of E. coli and associated virulence factors from sequence data. (A and B) For each sample the sequence data were mined to determine the percentage of reads mapping to E. coli H10407 (A) and the presence of adhesins and pathogenic E. coli-associated virulence factors (B). The intensity of the heatmap is shown relative to the RPKM for the gene of interest.

Regardless of age, participants who were symptomatically infected with ETEC were more likely to have high percentages of reads mapping to E. coli H10407 (Fig. 1A). The average percentage of reads that mapped to E. coli H10407 was also significantly greater in symptomatic participants compared to asymptomatically infected participants and ETEC-negative healthy controls. Asymptomatic participants had a higher average percentage of E. coli H10407 reads compared to controls (*P* = 0.002) but were significantly lower than the symptomatic group. While this suggests that symptomatically infected participants had a higher burden of ETEC on average, it is possible that they may also have had an abundance of other E. coli or related bacteria or that they were presymptomatic and would go on to have diarrheal symptoms.

To specifically identify ETEC and other diarrheagenic E. coli in the stool samples, pathotype-defining virulence gene sequences were detected in shotgun data using SRST2. ETEC-positive samples were defined as those where we could detect DNA sequences for either the heat-labile toxin (LT) or heat-stable toxin (ST) genes. Of the 15 symptomatic participants in the study, we could detect LT or ST in 14 samples (Fig. 1B). Importantly, the proportion of samples within which we could detect LT/ST and the reads per kilobase mapped (RPKM) for these genes was significantly greater in the symptomatically infected group compared to asymptomatically infected individuals (Wilcoxon rank-sum test, LT *P* = 0.0285; ST *P* = 0.0413). There was no significant difference in the detection of reads between adults and children.

In addition to ETEC-associated DNA, symptomatically infected participants had significantly higher levels of detectable DNA associated with enteroaggragative E. coli (EAEC) than asymptomatically infected participants (*P* = 0.003). DNA sequences normally associated with enteropathogenic E. coli (EPEC) were also detected in some samples, but there was no statistically significant difference between groups (Fig. 1B). Compared to control participants, symptomatically infected individuals had significantly higher detection of ETEC- and EAEC-associated DNA, while for asymptomatic participants, only ETEC-associated DNA signatures were significantly more prevalent (*P* = 0.009).

Interestingly, of the samples from which we could detect ETEC-associated DNA, the majority of symptomatic patients were infected with ETEC harboring genes for both LT and ST. In contrast, the most common ETEC found in asymptomatic patients was ST-only ETEC. ETEC positive for the LT gene only was only found in one patient in each infected group. Of the ETEC colonization factor adhesins identified, CFA and CS13 were significantly more prevalent in asymptomatic and symptomatic participants than controls (Fig. 1B). A number of additional adhesins were detected in symptomatic participants, including CS1, CS2, CS6, CS14, CS17, and CS22. Overall, the number of fimbriae detected per sample was higher in symptomatic participants than in those who were asymptomatic (Mann-Whitney U test, *P* < 0.01). This could be due to mixed ETEC infections or a higher average number of fimbriae produced by ETEC strains colonizing participants in the symptomatic group.

### Comparison of microbiome diversity and composition between asymptomatic and symptomatic individuals.

**FIG 2:**
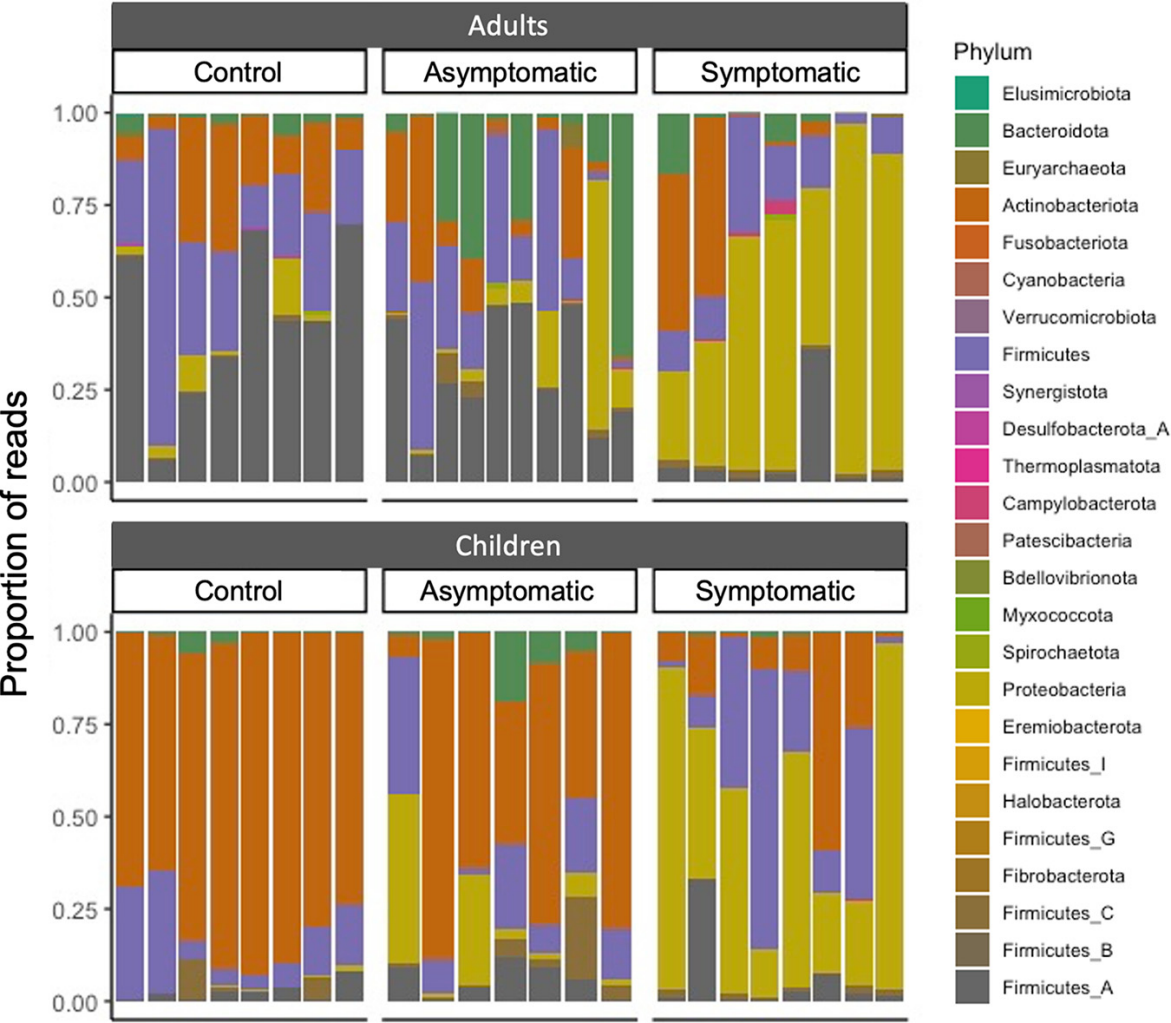
Microbial composition of gut microbiome samples by age group and health status. Sequenced reads were classified to the phylum level, and the relative abundance of each phylum is shown as the proportion of total bacterial reads.

To compare the gut microbiome composition of stool samples from ETEC-infected participants and controls, human sequence-depleted DNA reads were assigned informatically into taxonomic groups, and the microbial composition was compared at the phylum level (Fig. 2). As expected, there were noticeable differences between the microbial composition of stool between adults and children. In general, healthy adults were heavily colonized by *Firmicutes*, while healthy children were predominantly colonized by *Actinobacteria*. In contrast, symptomatic participants showed high proportions of *Proteobacteria*, regardless of age. While the majority of asymptomatic participants were similar to controls at the phylum level, there were some notable exceptions within the data set where asymptomatic participants looked more similar to symptomatic participants than to controls.

**FIG 3:**
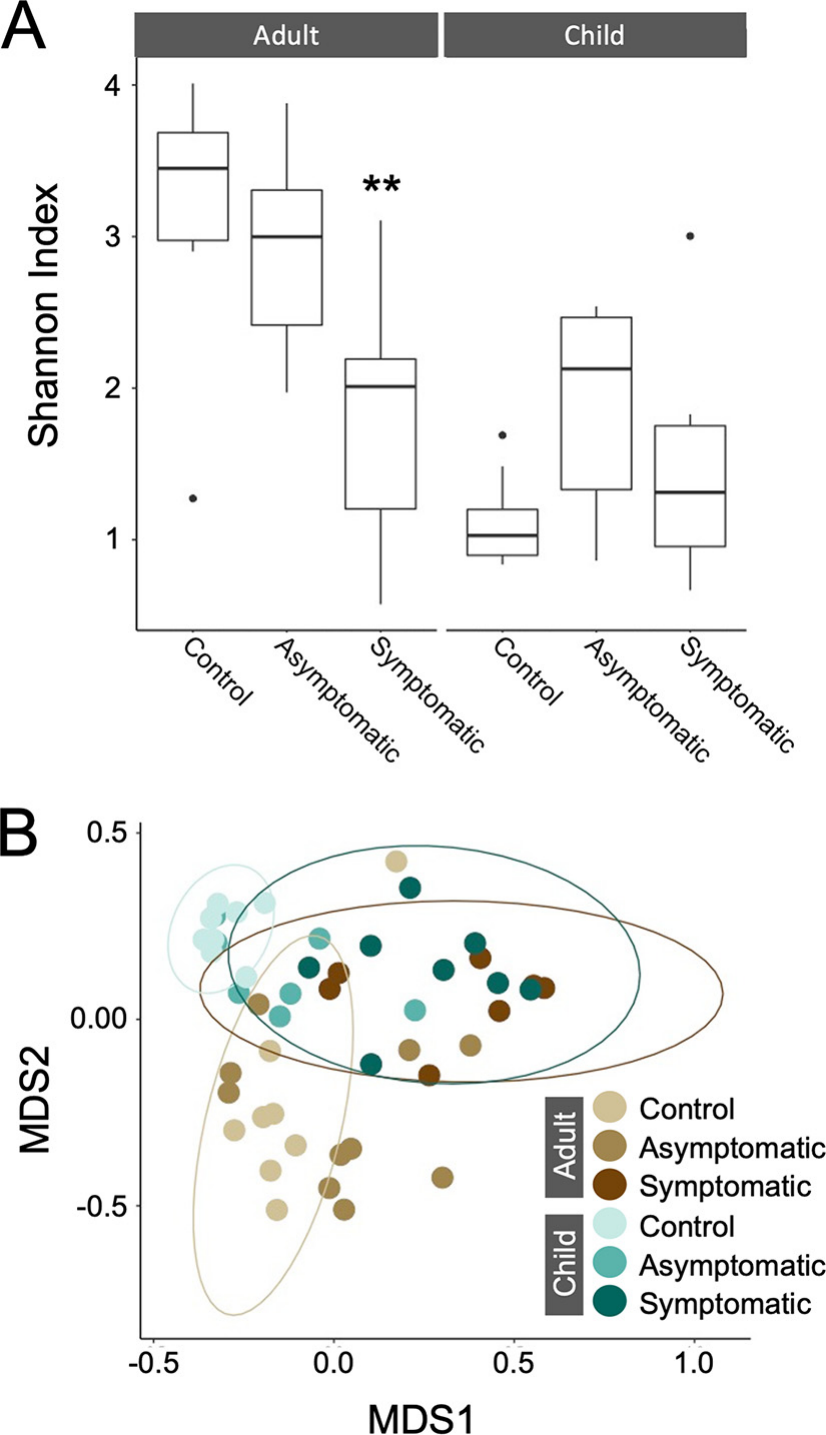
Diversity of stool microbiome samples by health status group and age. The diversity of microbiome samples was compared at the genus level and stratified by age group and health status. (A) Shannon (α) diversity (B) Bray Curtis dissimilarity (β diversity). MDS, multidimensional scaling; **, *P* < 0.01.

The differences in microbiome structure were further investigated with additional granulation by determining the richness (α-diversity) and composition (β-diversity) of the samples at the genus level. α-Diversity was measured using Shannon’s diversity index, a measure of population abundance and evenness. Higher Shannon indices are seen in samples which are more diverse in the number of species present and lower when the sample is populated by fewer species or predominantly composed of few species. As expected, the pattern of α-diversity was very different between adults and children (Fig. 3). The α-diversity of the microbiome in control and asymptomatic adults was significantly greater than that of symptomatic participants (Fig. 3A). In children however, the microbiome α-diversity in controls was found to be much lower than that of adult controls (Fig. 3A). There was a trend toward an increase in α-diversity in asymptomatic children compared to controls; however, this difference was not statistically significant. Symptomatic children had diversity profiles somewhere between controls and asymptomatic children but were not significantly different from either.

β-Diversity was compared by calculating the Bray Curtis dissimilarity index, which compares the compositional dissimilarity between samples. A Bray Curtis value of 0 reflects a situation whereby samples contained exactly the same microbes, whereas a value of 1 would suggest they did not share any microbial species. In general, symptomatically infected adults and children had similar microbial composition to each other, regardless of age (Fig. 3B, dark green and dark brown ellipses). Control adults and children had distinct microbial composition, as shown by the separate β-diversity populations (Fig. 3B, light green and light brown ellipses). Asymptomatically infected participants did not cluster together but were spread between the distinct control and symptomatic populations.

### Diversity of antimicrobial resistance genes between asymptomatically and symptomatically infected individuals and controls.

**FIG 4:**
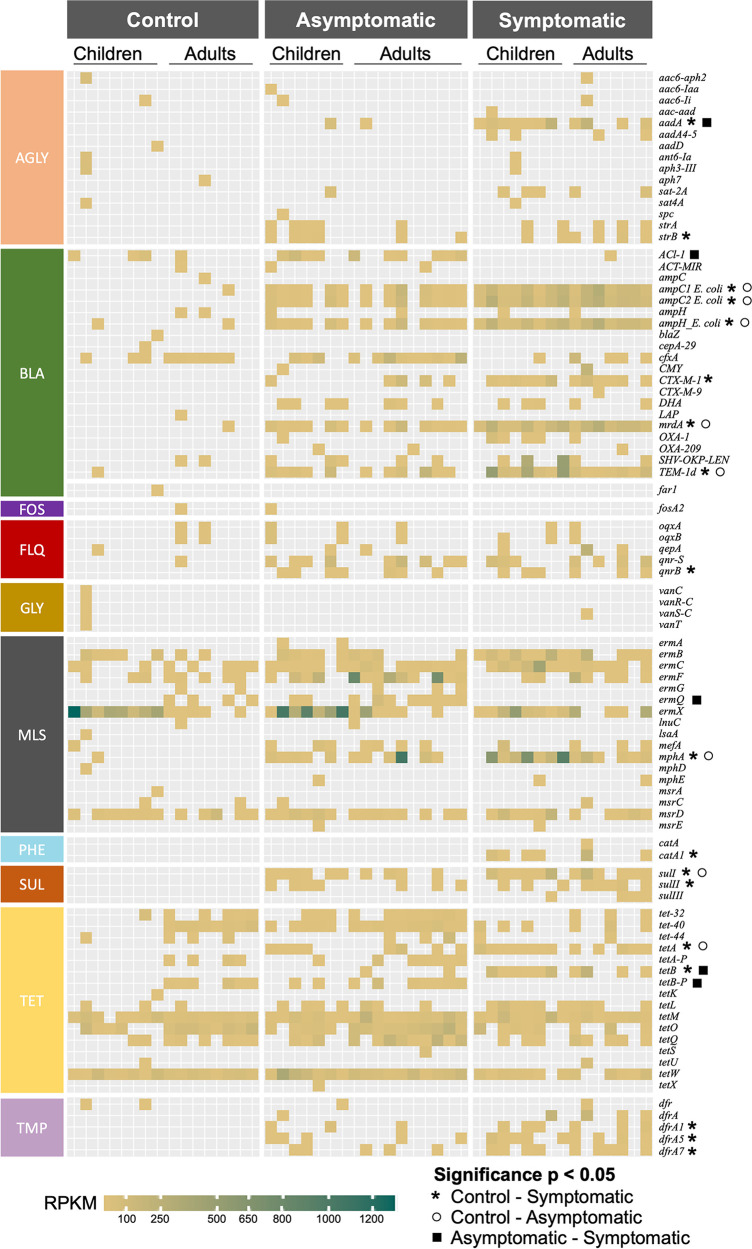
Antimicrobial resistance gene carriage in participants. RPKM values for ARGs detected in participant stool samples are shown as a heatmap, clustered by health status, age, and antimicrobial class. Significant differences (Fisher’s exact test) in the prevalence of specific ARGs between groups is shown next to the gene name. AGLY, aminoglycosides; BLA, beta-lactamases; FOS, fosfomycin; FLQ, fluoroquinolones; GLY, glycosides; MLS, macrolides/lincosamides/streptogramins; PHE, phenicols; SUL, sulfonamides; TET, tetracyclines; TMP, trimethoprim.

Antimicrobial resistance genes were identified from whole shotgun sequence data using SRST2 and the ARG-ANNOT database. To allow comparison between samples, the relative abundance of each gene in the shotgun data was determined by calculating the RPKM (Fig. 4). There were significant differences in the antibiotic genes detected in the different health status groups. In general, genes of the beta-lactamase, fluoroquinolone, sulfonamide, and trimethoprim classes were more common in symptomatic and asymptomatic patients than controls. Only *aadA* and *tetB* were significantly more prevalent in symptomatic patients than in asymptomatic patients. Conversely, *ACl-1* and *ermQ* were both found to be more prevalent in asymptomatic patients than controls. There were few remarkable differences between the age groups; however, in control participants a small number of tetracycline genes (*tet-40* and *tetB-P*) were more prevalent in adults than children.

### Differentially abundant taxa between individuals with high and low E. coli loads.

The above-described analysis of the microbiome samples showed significant heterogeneity between asymptomatically infected participants. It is clear, however, that despite ETEC exposure, some individuals were able to protect themselves from an outgrowth of E. coli, while others became heavily colonized. To further explore the differences in the microbiomes of people who were highly colonized with ETEC compared to those with low or no ETEC colonization, we reclassified samples into two categories, those with high E. coli burden (>10% reads mapped to E. coli H10407) and those with low E. coli burdens (<10% reads mapped to E. coli H10407). For adults, 9/25 (36%) of enrolled participants had high proportions of E. coli reads, while for children this was 10/23 (43%).

**FIG 5:**
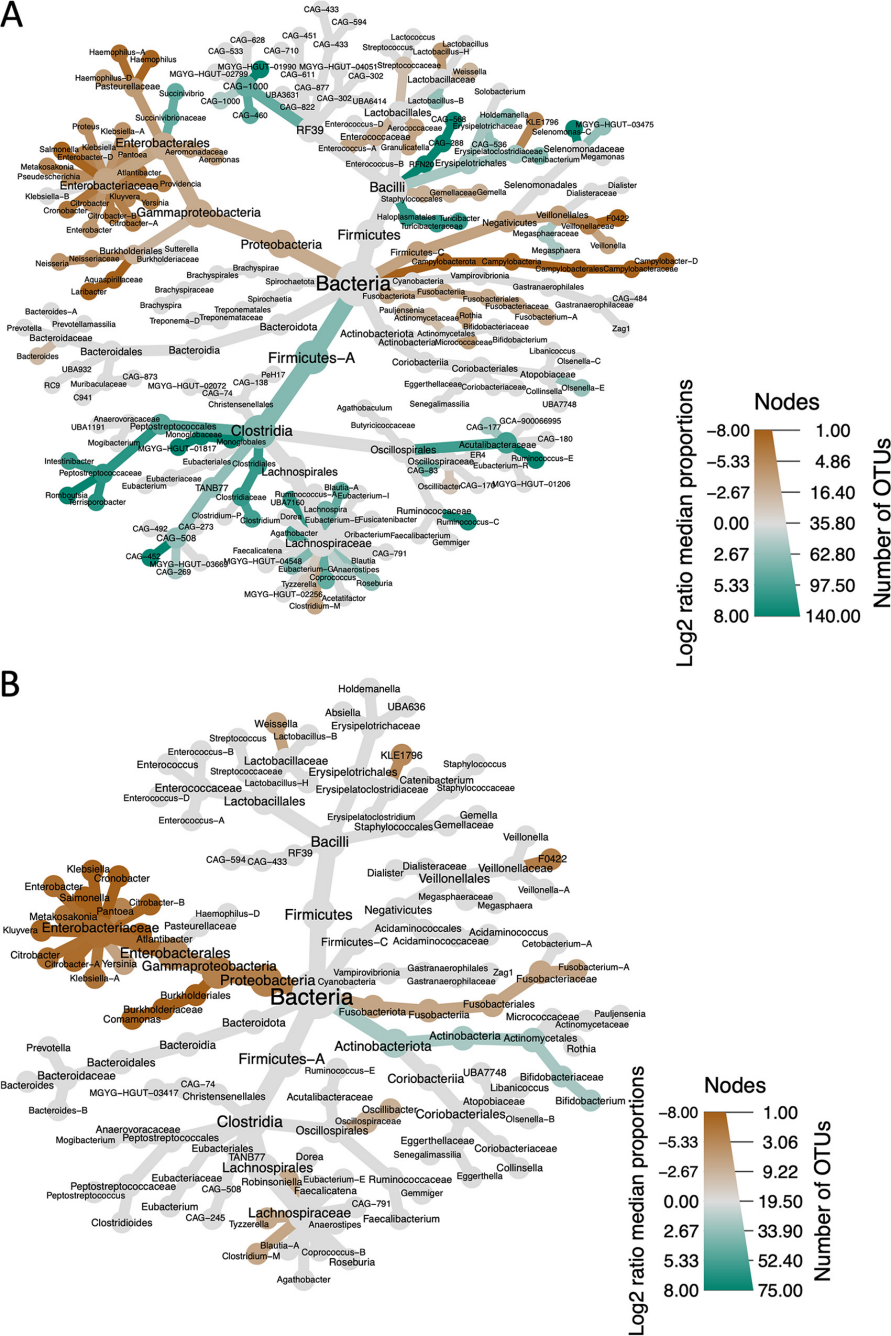
(A and B) Heat trees showing statistically significant differences in abundance of bacterial taxa in adults (A) and children (B) with high (brown) or low (green) E. coli burden. The size of the node is proportional to the number of operational taxonomic units (OTUs) detected, while the depth of color is proportional to the size of the log_2_ median difference between groups. Gray branches show taxa that were present but not significantly different between groups.

To allow us to identify underlying differences in the non-E. coli bacterial populations in participants infected with ETEC, the reads classified as genus Escherichia were removed from the data set before analysis. For comparison between groups, abundance data at the genus level were normalized, and pairwise comparisons between each group were made by calculating the log_2_ ratio of median proportions. The median ratios were then filtered to remove hits that were not significantly different between groups. The significant differences between the high- and low-E. coli burden groups were then visualized using heat tree maps (Fig. 5). To provide additional granularity, this analysis was repeated at the species level (Table S1).

10.1128/mbio.00157-22.1 TABLE S1 Significant differences in bacterial abundance between samples with high E. coli burden and those with low E. coli burden at the species level. A positive log2 median ratio represents species that were overabundant in low-E. coli burden subjects, while a negative ratio indicates bacteria more prevalent in high-E. coli samples. Download Table S1, XLSX file, 0.02 MB. Copyright © 2022 Higginson et al. 2022 Higginson et al. https://creativecommons.org/licenses/by/4.0/ This content is distributed under the terms of the Creative Commons Attribution 4.0 International license.

There were 101 and 43 species significantly different in abundance in adults and children, respectively. Adults and children with high E. coli burdens had higher proportions of numerous other *Enterobacteriaceae* compared to those with low E. coli burdens. At the species level this included Salmonella enterica, *Citrobacter* spp., and Klebsiella spp. Adults with high E. coli reads also harbored higher proportions of reads that mapped to the genomes of *Campylobacteria*, *Burkholderiales*, *Pasteurellaceae*, and undefined *Veillonellaceae* F0422 MGYG-HGUT-03197 (Fig. 5A). In contrast, adults with low E. coli burden had significantly higher proportions of numerous as yet undefined species, including members of the classes *Bacilli* and *Clostridia*.

For children, there were fewer statistically significant differences between the two groups, and fewer taxa that contributed greater than 0.005 reads to the population, proportionally (Fig. 5B). In addition to members of the *Enterobacteriaceae*, other species that were highly enriched in children with high E. coli reads were Streptococcus spp., *Comamonas* spp., and the newly defined species F0422 MGYG-HGUT-03197, which was also identified in adults. The only taxa that were significantly enriched in children with low E. coli burden were six *Bifidobacterium* species (*B. infantis*, *B. scardovii*, *B. pullorum*, *B. gallinarum*, B. breve, and *B. angulatum*).

## DISCUSSION

In this study, we found significant differences in microbial diversity among symptomatically and asymptomatically ETEC-infected individuals compared to controls in both adults and children. The most striking difference between symptomatic and asymptomatic participants was the number of reads mapping to the widely used reference ETEC H10407 genome. Although our analysis may not provide an exact measure of ETEC burden, as it includes other E. coli, the E. coli read depths largely correlated with detected LT and ST toxin genes, which are key signatures of ETEC. This strongly suggests that asymptomatic people have a lower ETEC burden than symptomatic individuals. In this proof-of-concept study, asymptomatic participants were substantially more likely to harbor ST-only ETEC, whereas more symptomatic participants had LT/ST-ETEC. However, why ETEC is able to persist and replicate in some individuals without causing clinical disease remains an open question. While the presence of LT could increase the likelihood of a patient becoming symptomatic, data from large-scale studies suggest that ST is the more important toxin for mediating moderate-to-severe diarrhea (2).

One limitation of this small, proof-of-concept study was that samples were only collected from participants at one time point, and participants with detectable ETEC in their stool were not followed up to establish whether or not a subset would eventually go on to become symptomatic. While progression to symptomatic infection cannot be discounted within this cohort, we do know from case-control studies such as GEMS1 and GEMS1A that ETEC (LT or ST positive) is commonly identified in asymptomatic individuals (2, 15). From the GEMS1 study in Bangladesh, only ST-positive ETEC was significantly associated with disease, although this was only in the younger age groups. In children between 24 and 59 months, all-cause ETEC (ST or LT positive) was detected in 20 cases and 26 controls (https://clinepidb.org/ce/app/). Thus, there is a clear case for asymptomatic carriage of ETEC in children and adults. Building on this work, a larger study collecting longitudinal samples from participants will be needed to investigate how the microbiome changes in response to ETEC colonization over time and what factors precipitate progression to symptomatic disease.

There were no significant differences between adults and children in the E. coli read depth, ETEC toxin gene carriage, ARG carriage, or presence of other E. coli pathotypes. However, the microbiome diversity and response to infection were considerably different between these two groups. Microbial diversity in control children was relatively low, and their microbiomes were dominated by *Actinobacteria*. The diversity in control children was distinct from that of asymptomatically infected children, who had much higher α-diversity. This likely represents the proliferation of species normally present at low abundances in these children due to disruption of normal gut homeostasis. Whether ETEC infection precipitates this change or is a result of it is yet to be determined. In general, when looking at microbial diversity, the microbiomes of symptomatic and asymptomatic children were more similar to each other than to controls. This is an important factor to consider when deciding on appropriate controls for future microbiome research or potential microbial therapeutic development.

On the species level we also saw increases in the proportions of several *Streptococcal* species in children with high reads. This is consistent with results of a similar study on microbiome changes during intestinal infection in Bangladesh, which found proportional increases in E. coli and several *Streptococcal* species in children with diarrhea, regardless of the culpable intestinal pathogen (6). Conversely, in children with low E. coli burden we found higher proportions of several species of *Bifidobacterium*. These bacteria are common in breastfed infants (16) and were also increased in abundance in healthy children compared to diarrhea cases in the above-described study (7).

For adults, the overall microbiome profile was very different from that in children. Asymptomatic adults and controls had very similar gut microbiomes, which suggests that the introduction of ETEC does not singularly cause any considerable microbiome perturbation and that there may be other factors at play. The healthy adult microbiome was also much more diverse, indicating decreased diversity upon ETEC infection as E. coli became the most abundant bacterial member of the microbiome. The microbiome in healthy adults was rich in *Firmicutes*, similar to other published studies from Bangladesh (14), although we also observed an abundance of *Actinobacteria*. This finding was similar to a study on travelers returning to the United States from a LMIC, which found an abundance of *Firmicutes*, *Actinobacteria*, and *Proteobacteria* in healthy and ETEC-infected travelers (17). Although a study of ETEC infections in healthy volunteers in the United States found a number of *Bacteroides* species to be protective against infection (14), in our study the *Bacteroides* genus was on average greater than 2-fold more abundant in adults with high E. coli sequence reads, although no species were significantly more abundant. This may be related to baseline differences in gut microbiome composition between the United States and Bangladesh.

It is clear from these data that progression to symptomatic ETEC infection is associated with an outgrowth of *Enterobacteriaceae*, although interestingly, many uncharacterized species were also significantly associated with either protection from or progression toward symptomatic disease. Most of these bacteria have only recently been discovered, and many have never been cultured, as they were identified through metagenome assembly. This highlights the importance of efforts like the Unified Human Gastrointestinal Genome to shine a light on previously hidden microbial populations (17).

Finally, ARG carriage was significantly different between the groups, with asymptomatic and symptomatic patients being broadly more similar to each other than controls. In general, patients colonized with ETEC had higher prevalence of ARGs of the trimethoprim, sulfonamide, fluoroquinolone, and beta-lactamase classes. These antibiotics are commonly used in South Asia, both clinically and in farming, and high levels of resistance to these classes have been seen in other studies (18–21). Although there was increased prevalence of ARGs in people colonized with ETEC, it is unlikely that all the ARGs would be ETEC-associated, as most E. coli isolates in South Asia are not so highly multidrug resistant (18, 19). It is therefore probable that the increase in ARG prevalence is associated with changes in the microbiome and increases in the relative proportions of ARG-carrying *Enterobacteriaceae*. Understanding the mechanics of this interaction will require larger studies encompassing longitudinal sampling and comprehensive lifestyle and antibiotic usage surveys.

### Conclusions.

Our study highlights the gastrointestinal microbiome differences between asymptomatic colonization with ETEC compared to symptomatic infection. Not only were there clear alterations in microbial composition in these patients, but the prevalence of several clinically relevant ARG classes was also impacted. This work provides important foundational information for future studies investigating the impact of gut microbiome composition on gastrointestinal disease progression and how the presence of supportive organisms may endow protection against symptomatic disease.

## MATERIALS AND METHODS

### Ethics.

Samples were collected from participants of the ETEC ETVAX Vaccine Trial (ClinicalTrials registration no. NCT02531802) at the International Centre for Diarrhoeal Disease Research, Bangladesh (icddr,b).

### Sample collection.

Stools samples were collected from asymptomatic and symptomatic adults and children who were culture positive for ETEC, as well as uninfected controls (Table 1). Raw stool was stored at −80°C before total DNA was extracted using the QIAamp DNA stool minikit. All samples were processed within 4 days of sample collection.

**TABLE 1:** Numbers of study participants by age and health status

Age group	Control	Asymptomatic	Symptomatic	Total
Children	8	7	8	23
Adults	8	10	7	25
Total	16	17	15	48

### Sequencing.

DNA samples that cleared the quality-control check were sequenced in 16-plex on two lanes of a HiSeqV4 machine at the Wellcome Sanger Institute (WSI). The raw data are available on the European Nucleotide Archive under study accession no. PRJEB21793. Over 15 million 125-bp paired-end reads were generated for each sample. The data generated were screened for human reads, which were identified and removed from the raw sequence data by aligning to the Homo sapiens GRCh38 reference genome using Bowtie 2 v2.2.3 (22).

### E. coli mapping, virulence factor, and ARG detection.

To identify E. coli species-specific reads in raw sequence data, human-depleted reads were mapped to the widely used O78 reference genome of H10407 (accession version no. GCA_000210475.1) using Bowtie 2. To identify relevant E. coli virulence determinants and ARGs, raw reads were mapped against databases of E. coli virulence determinants (Virulence Factor Database [23]) and ARGs (ARGannot3 [REF]) using SRST2 v2 (24). The number of reads mapping to these genes of interest was quantified by calculating the reads per kilobase mapped (RPKM). Pathotypes were defined as follows: enterotoxigenic Escherichia coli (ETEC) were defined as the presence of the genes corresponding to heat-labile toxin (LT) or heat-stable toxin (ST), enteroaggregative Escherichia coli (EAEC) were defined by the presence of *aatA* and at least one of either the *aggR*, *aaiC*, or *aap* genes, and enteropathogenic Escherichia coli (EPEC) were defined as *eae* positive and *stx* negative.

Statistical analysis of pathotype detection was calculated using a two-tailed Fisher’s exact test, and the percentage of mapped reads and RPKM (reads per kilobase of transcript, per million reads mapped) values were compared using a Wilcoxon rank-sum test. To compare the prevalence of antibiotic resistance gene detections between groups, Fisher’s exact test was used with an adjustment for false-discovery rate.

### Taxonomic classification, abundance estimation, and diversity analyses.

Classification of human-depleted reads was carried out using Kraken 2 v2.0.8-beta (25). Reads were classified against the uhgg_kraken2-db database of prokaryotic genomes (17), which was downloaded from the European Bioinformatics Institute (https://www.ebi.ac.uk/metagenomics/genomes). Abundance estimation was run on Kraken 2 outputs using Bracken v2.5 (26) at the phylum and genus levels. Diversity analyses were carried out in R using vegan v2.5-6 (27) and agricolae v1.3-1 (28). Before α- and β-diversity calculations, data were rarefied to the minimum number of reads to maintain all samples in the data set. Statistical analysis of α-diversity measures was carried out using analysis of variance (ANOVA) and Tukey’s honestly significant difference (HSD) test.

Metacoder v0.3.3 (29) was used to visualize genus level differences in bacterial diversity between samples with high (>10%) and low percentages (<10%) of reads mapping to E. coli H10407. Bracken abundance data were filtered to limit the data set to bacterial taxa. To discount the contribution of Escherichia reads on the overall abundance of taxa, reads classified to the level of Escherichia were removed from the data set. Due to the differences in diversity seen between adults and children, these data sets were analyzed independently. Low-abundance taxa (<20 reads per sample) were removed, and proportions of total bacterial reads were calculated for the remaining taxa. Taxa with proportions below 0.005 for all samples were dropped from the analysis. For comparative analysis, the log_2_ median ratio of taxon abundances was calculated for each pairwise comparison. Statistical significance between groups was measured using the Wilcoxon rank sum test, with an adjustment for false-discovery rate. For visual heat tree comparisons, log_2_ median ratios were filtered to display only those that were significantly different between groups.

### Data availability.

The sequence data are available on the European Nucleotide Archive under study accession no. PRJEB21793.
